# The American Public Health Association

**Published:** 1882-02

**Authors:** 


THE AMERICAN PUBLIC HEALTH ASSOCIATION.—In our last issue we promised to recur to the presidential address of Dr. White before the American Public Health Association, with a view of discussing some of the problems that meet us in the consideration of the question: "What constitutes sanitary science?" Dr. White has however discussed the whole subject in such an interesting manner, that we refer our readers to the address itself, which we publish in part in our *Popular Science Department.*

It will well repay perusal for its statements of fact and its keen criticisms of many of our sanitary methods and theories, as well as for the witty and striking manner in which his suggestions are offered. Dr. White is one of the *practical* sanitarians of the country, and in his work in New Orleans, both as president of the Board of Health, and more recently as medical director of the Auxiliary Sanitary Association, has manifested organizing and executive abilities of a very high order. In his discussion of the important problems involved, he is on familiar ground, and there are few who will not be instructed by the reading of his excellent address.

